# Characterization of a novel KCNQ1 mutation for type 1 long QT syndrome and assessment of the therapeutic potential of a novel IKs activator using patient-specific induced pluripotent stem cell-derived cardiomyocytes

**DOI:** 10.1186/s13287-015-0027-z

**Published:** 2015-03-19

**Authors:** Dongrui Ma, Heming Wei, Jun Lu, Dou Huang, Zhenfeng Liu, Li Jun Loh, Omedul Islam, Reginald Liew, Winston Shim, Stuart A Cook

**Affiliations:** National Heart Research Institute Singapore, National Heart Centre Singapore, 5th Hospital Drive, Singapore, 169609 Singapore; Cardiovascular & Metabolic Disorders Program, Duke-NUS Graduate Medical School Singapore, 8 College Road, Singapore, 169857 Singapore; National Heart and Lung Institute, Imperial College, South Kensington Campus, London, SW7 2AZ UK

## Abstract

**Introduction:**

Type 1 long QT syndrome (LQT1) is a common type of cardiac channelopathy associated with loss-of-function mutations of *KCNQ1*. Currently there is a lack of drugs that target the defected slowly activating delayed rectifier potassium channel (IKs)*.* With LQT1 patient-specific human induced pluripotent stem cell (hiPSC)-derived cardiomyocytes (hiPSC-CMs), we tested the effects of a selective IKs activator ML277 on reversing the disease phenotypes.

**Methods:**

A LQT1 family with a novel heterozygous exon 7 deletion in the *KCNQ1* gene was identified. Dermal fibroblasts from the proband and her healthy father were reprogrammed to hiPSCs and subsequently differentiated into hiPSC-CMs.

**Results:**

Compared with the control, LQT1 patient hiPSC-CMs showed reduced levels of wild type *KCNQ1* mRNA accompanied by multiple exon skipping mRNAs and a ~50% reduction of the full length Kv7.1 protein. Patient hiPSC-CMs showed reduced IKs current (tail current density at 30 mV: 0.33 ± 0.02 vs. 0.92 ± 0.21, P < 0.05) and prolonged action potential duration (APD) (APD 50 and APD90: 603.9 ± 39.2 vs. 319.3 ± 13.8 ms, P < 0.005; and 671.0 ± 41.1 vs. 372.9 ± 14.2 ms, P < 0.005). ML277, a small molecule recently identified to selectively activate K_V_7.1, reversed the decreased IKs and partially restored APDs in patient hiPSC-CMs.

**Conclusions:**

From a LQT1 patient carrying a novel heterozygous exon7 deletion mutation of *KCNQ1*, we generated hiPSC-CMs that faithfully recapitulated the LQT1 phenotypes that are likely associated with haploinsufficiency and trafficking defect of *KCNQ1*/Kv7.1. The small molecule ML277 restored IKs function in hiPSC-CMs and could have therapeutic value for LQT1 patients.

**Electronic supplementary material:**

The online version of this article (doi:10.1186/s13287-015-0027-z) contains supplementary material, which is available to authorized users.

## Introduction

Long QT syndrome (LQTS) are inherited arrhythmic heart diseases characterized by prolonged QT intervals on electrocardiograms (ECGs) and sudden cardiac deaths. To date, the understanding of mutation-dependent disease mechanisms and the development of evidence-based clinical therapies for LQTS have been hampered by a lack of ideal disease models that enable precise analysis of disease characteristics. The recent breakthrough in generating human induced pluripotent stem cells (hiPSCs) from postnatal humans and their ability to be differentiated into functional cardiomyocytes (hiPSC-CMs) have opened a new page in the study of inherited cardiac diseases such as LQTS. To date, patient-specific hiPSC lines have been successfully generated from individuals with types 1, 2, 3 and 8 (Timothy syndrome) LQTS and cardiomyocytes derived from these lines, as *in vitro* models, have faithfully recapitulated cardinal cellular disease phenotypes [1-4].

Type 1 long QT syndrome (LQT1) is the most common subtype of LQTS (~40% of all LQTS) that is associated with loss of function of the slowly activating delayed rectifier potassium channel (IKs) in cardiomyocytes [5-7]. The IKs current is mediated by the voltage-gated potassium channel Kv7.1, which consists of the α-subunit (encoded by *KCNQ1*) that forms heterodimers with β-subunits (MinK, encoded by *KCNE1*). The actual ion channel is made up of four α-subunits. The exon 6 to exon 7 junction of *KCNQ1* represents a splicing mutation hotspot and contains several splicing mutations [8-11].

Using patient-specific hiPSC-CMs as a disease model, this study aimed to characterize the impacts of a novel *KCNQ1* mutation (heterozygous deletion of exon 7) identified in a LQT1 patient and to evaluate the therapeutic potential of ML277 in LQT1 hiPSC-CMs.

## Materials and methods

For detailed procedures, please refer to Methods in Additional file 1.

### Patient recruitment

**Figure 1:**
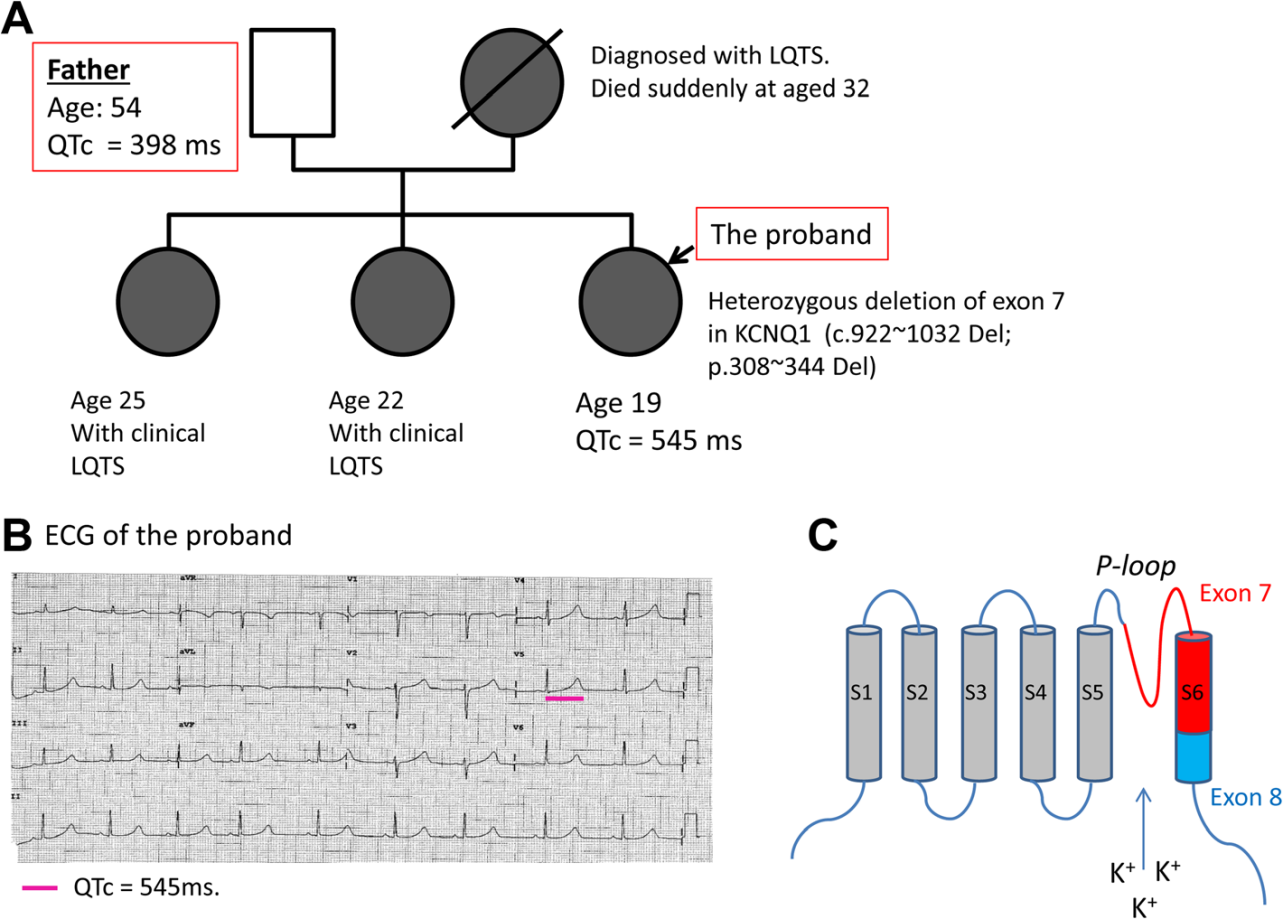
**Data for the type 1 long QT syndrome family. (A)** Family pedigree. **(B)** Electrocardiogram (ECG) of the type 1 long QT syndrome patient represents prolonged QT intervals. **(C)** Cartoon showing the position of exon 7-encoded pore region in the α-subunit of K_V_7.1. LQTS, long QT syndrome; QTc, corrected QT interval.

The study was approved by the SingHealth institutional review board (Singapore) following principles in the Declaration of Helsinki. Clinical data as well as blood and skin samples were taken following written informed consent. A family of LQT1 was identified based on the clinical symptoms and ECG (Figure 1). The proband (the LQT1 patient) and her clinically normal father (the family control) (Figure 1A) participated in this study. The ECG of the LQT1 patient (Figure 1B) showed typical prolongation of QT interval (corrected QT interval = 545 ms) (Figure 1B). The ECG recorded from the father (not shown) was normal (corrected QT interval = 398 ms).

### Generation and characterization of human induced pluripotent stem cells

Dermal fibroblasts of the patient and the control were established from the dermis tissue obtained from a 5 mm punch skin biopsy. Fibroblasts were reprogrammed to hiPSCs via retroviral transduction of Yamanaka transcription factors OCT-4, SOX2, KLF4 and c-MYC [12,13]. The pluripotency of hiPSCs was confirmed by their expression of pluripotent stem cell markers determined by immunofluorescence assay and further validated by teratoma formation assay. Moreover, karyotyping was performed to check the genomic stability of hiPSCs.

### Cardiac differentiation of human induced pluripotent stem cells

Cardiac differentiation of hiPSCs was achieved via Wnt signaling inhibition of a monolayer of hiPSCs formed from single cells [14].

### iCell Cardiomyocyte

iCell Cardiomyocyte from Cellular Dynamics International Inc. (Madison, WI, USA), hereby called iCell, is a normal hiPSC-CM line [15] adopted in this study as an additional control for evaluating the effects of the ML277.

### Gene expression of *KCNQ1*

Total RNA was isolated from hiPSC-CMs and reverse transcribed into cDNA with Superscript III Reverse Transcriptase (Invitrogen, Singapore, Singapore). The expression of the *KCNQ1* gene in hiPSC-CMs was measured by semi-quantitative RT-PCR assay. The PCR products covering exon 6 to exon 9 of the *KCNQ1* gene (primers shown in Table S1 in Additional file 1) were gel-purified and sequenced.

### Total levels of Kv7.1 and its intracellular localization

The total cellular K_V_7.1 level was determined by western blot. Cell lysate was prepared from the clusters of hiPSC-CMs and incubated with primary antibody Anti-K_V_7.1. The intracellular localization of K_V_7.1 in hiPSC-CMs was determined by immunofluorescence assay with primary antibodies including anti-K_V_7.1, anti-α-actinin and anti-Golgi. The fluorescent intensity was quantified with ImageJ software (National Institutes of Health, Bethesda, MD, USA), where signals with a distance to the nucleus less than one-half of the radius were marked as perinuclear and the rest marked as membranous.

### Whole cell patch-clamp recordings

Dissociated hiPSC-CMs 4 to 5 weeks post cardiac differentiation were plated into 3.5 cm Petri dishes coated with gelatin (0.1%, w/v). Whole cell patch-clamp recordings were conducted with an Axon patch 200B amplifier controlled by Axon Instruments pClamp10 software via the Digidata 1440 acquisition system software (Molecular Devices, LLC., Sunnyvale, CA, USA). Cardiac action potentials (APs) were recorded with current-clamp protocols of standard whole cell patch-clamp techniques [4]. The IKs currents were recorded with voltage-clamp protocols of standard whole cell patch-clamp techniques [16].

ML277, or (R)-*N*-(4-(4-methoxyphenyl)thiazol-2-yl)-1-tosylpiperidine-2-carboxamide, was purchased from Sigma-Aldrich Corp. (St Louis, MO, USA).

### Statistical analyses

Numerical data are presented as the mean ± standard deviation, except for the electrophysiological data that are presented as mean ± standard error of the mean. Comparisons were performed with an unpaired Student *t* test (two-tailed) and one-way analysis of variance followed by Tukey’s post test. Pearson’s chi-squared test was used to compare the proportions of wild type (WT) and various exon-skipping mRNAs. *P* <0.05 was considered statistically significant.

## Results

### A novel *KCNQ1* mutation was identified in the LQT1 family

An in-frame heterozygous deletion of exon 7 (*C*.922 ~ 1,032 del; *P.*308 ~ 344 del) in the *KCNQ1* gene, which was not previously reported in LQT1, was identified in the proband and her mother and sisters (Figure 1A). Exon 7 encodes a major part of the S6 transmembrane-spanning segments (the pole region) and the loop connecting S5 and S6 (Figure 1C). No pathogenic mutations of up to 50 other cardiac genes (including *KCNE1*) responsible for inherited heart diseases were identified.

### LQT1 patient-specific hiPSCs and hiPSC-CMs were generated

LQT1 patient and control hiPSCs were derived and their pluripotency was confirmed *in vitro* and *in vivo* by the expressions of typical pluripotent stem cell markers (Figure S1A in Additional file 1) and formation of teratoma (Figure S1B in Additional file 1), respectively. The hiPSCs maintained a normal karyotype (Figure S1C in Additional file 1).

### LQT1 patient-specific hiPSC-CMs express reduced WT *KCNQ1* accompanied by alternative transcriptions skipping exon 7 and exon 7 plus exon 8

**Figure 2:**
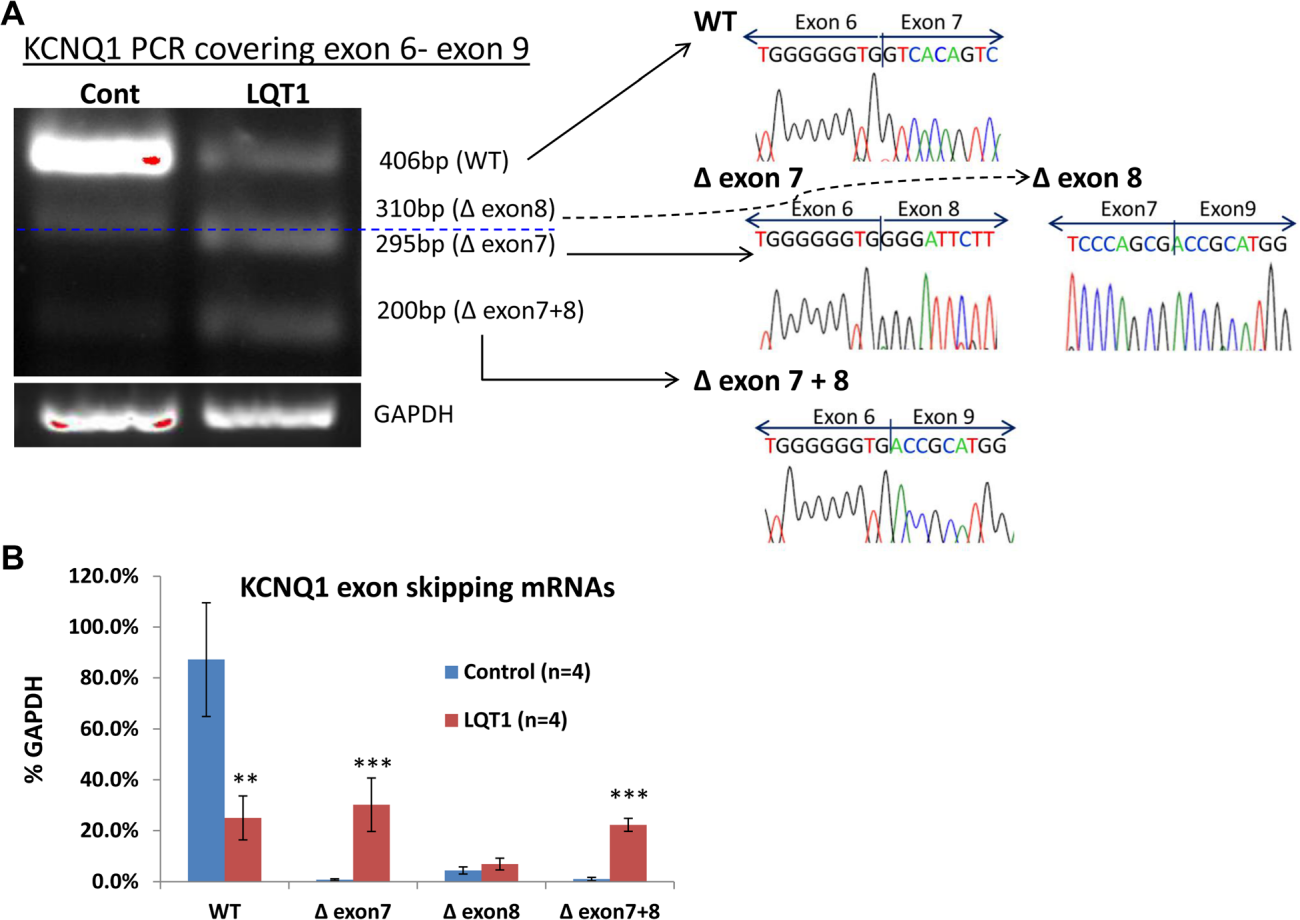
**Expression of the** ***KCNQ1*** **gene in LQT1 patient-derived human induced pluripotent stem cell cardiomyocytes. (A)** (Left) RT-PCR images covering *KCNQ1* cDNA from exon 6 to exon 9 in control and patient human induced pluripotent stem cell cardiomyocytes (hiPSC-CMs). Blue dashed line separates the PCR products of ∆exon8 (above) from ∆exon7 (below). (Right) Sequences of the corresponding PCR products. **(B)** Quantitative profiling of the wild type (WT) and exon-skipping mRNAs in control and patient hiPSC-CMs. Data presented as mean ± standard deviation. **P <0.01, ***P <0.001, versus control. bp, base pairs; GAPDH, glyceraldehyde 3-phosphate dehydrogenase; LQT1, type 1 long QT syndrome.

hiPSCs were differentiated into hiPSC-CMs. By sequencing of the PCR products, alternative transcriptions of *KCNQ1* in LQT1 patient hiPSC-CMs were confirmed. Compared with the control, LQT1 patient hiPSC-CMs showed a markedly reduced expression level of the WT *KCNQ1* gene, which was accompanied by a *de novo* alternative transcription isoform skipping exon 7 (Δexon7) (Figure 2). In addition, a significant increment of an exon 7 plus exon 8 skipping (Δexon 7 + 8) transcription was detected in patient hiPSC-CMs. It was noted that in control hiPSC-CMs a small amount of Δexon8 alone and Δexon7 + exon8 isoforms was detectable (Figure 2).

Our data indicate that the heterozygous ∆exon 7 in *KCNQ1* led to decreased WT *KCNQ1* transcription, a *de novo* ∆exon 7 isoform and a significant increment of the Δexon7 + exon8 isoform.

### LQT1 patient-specific hiPSC-CMs expressed reduced levels of WT Kv7.1 protein

**Figure 3:**
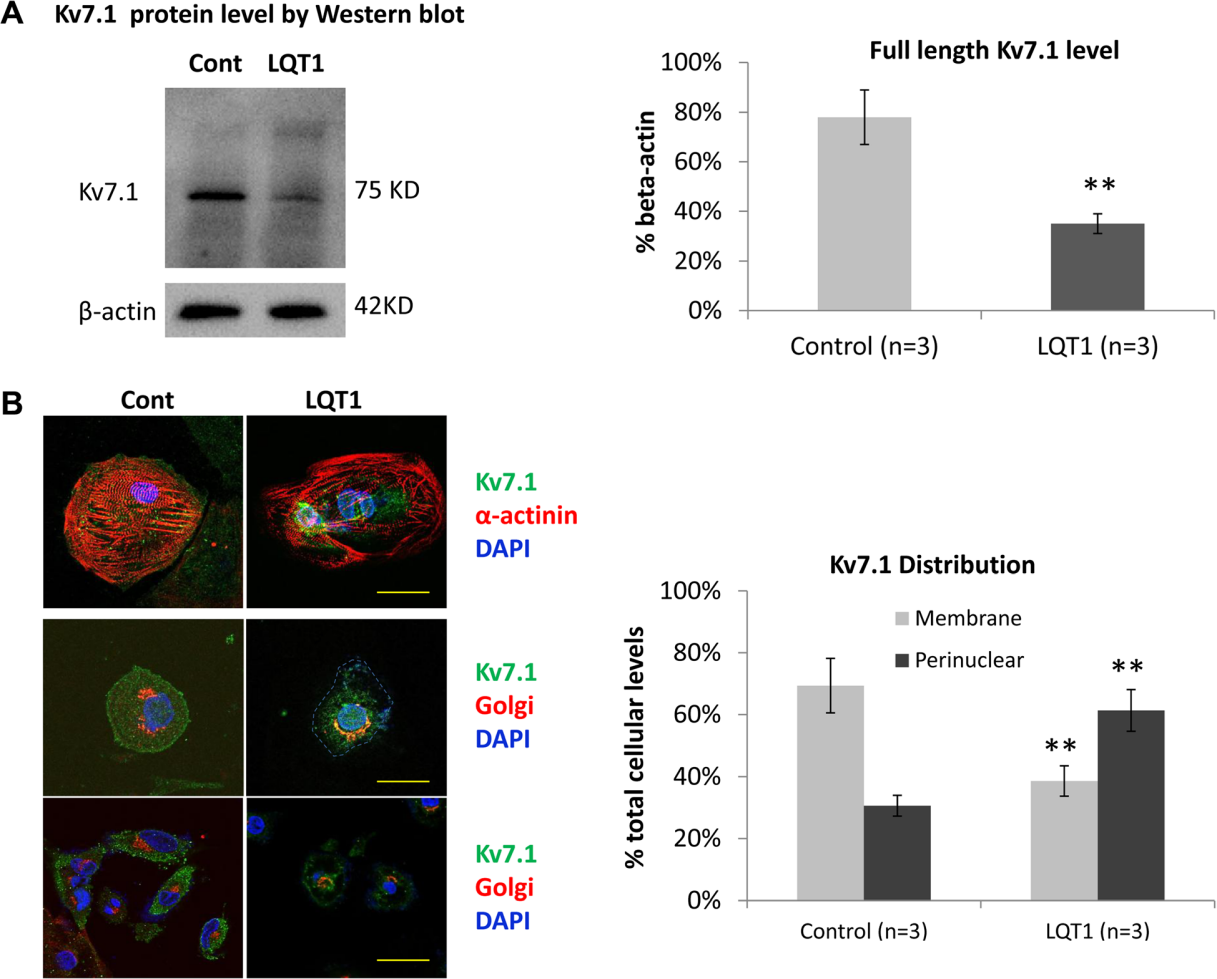
**Total levels and intracellular distributions of K**_**V**_ **7.1 in LQT1 patient-derived human induced pluripotent stem cell cardiomyocytes. (A)** Representative western blot image of Kv7.1 (left) and bar graft showing the levels of K_V_7.1 plotted against β-actin (right). **P <0.01, versus control. **(B)** Representative images of co-immunofluorescence staining of α-actinin (red) and K_V_7.1 (green) (left top panel, 63×; scale bar: 20 μM) and Golgi apparatus (red) and Kv7.1 (green) (left middle panel, 63×; scale bar: 20 μM; and left bottom panel, 40×; scale bar: 40 μM) in control and patient human induced pluripotent stem cell cardiomyocytes (hiPSC-CMs). Cell nuclei were stained with 4',6-diamidino-2-phenylindole (DAPI; blue). The membranous and perinuclear distributions of K_V_7.1 were quantified in K_V_7.1 and Golgi apparatus co-staining images by ImageJ software (National Institutes of Health, Bethesda, MD, USA). The relative ratio was plotted and presented in a bar graph (right). **P <0.01, versus control. LQT1, type 1 long QT syndrome.

A significant reduction of the total cellular level of the full length (WT) α-subunit of Kv7.1 potassium channel (~75 kDa) was observed in LQT1 patient-derived hiPSC-CMs compared with the control. The ratios of the full length Kv7.1 normalized to β-actin in the control and patient hiPSC-CMs (mean ± standard deviation) were 54 ± 9% and 33 ± 4% (*P* <0.01). However, the protein isoforms predicted based on exon skipping in the α-subunit of Kv7.1 were largely not detectable (Figure 3A).

The immunofluorescence staining of Kv7.1 showed a perinuclear localization pattern in patient hiPSC-CMs compared with a perimembrane distribution pattern in the control (Figure 3B). The membrane and perinuclear staining of Kv7.1 in patient versus control was 38.6% and 61.4% versus 69.4% and 30.6% (*P* <0.001).

Taken together these data indicate possible haploinsufficiency and trafficking defect of *KCNQ1*/Kv7.1 in patient cardiomyocytes.

### LQT1 patient-specific hiPSC-CMs demonstrated decreased IKs and prolonged action potential duration

**Figure 4:**
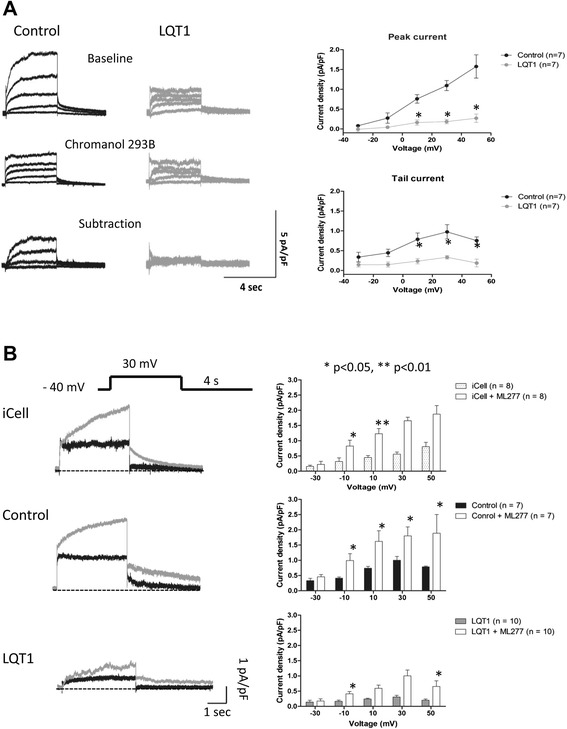
**Slowly activating delayed rectifier potassium channel measured in human induced pluripotent stem cell cardiomyocytes. (A)** Slowly activating delayed rectifier potassium channel (IKs) currents recorded in patient and control human induced pluripotent stem cell cardiomyocytes (hiPSC-CMs). IKs currents were validated as Chromanol 293B-sensitive IKs currents (after subtraction). Averaged peak and tail current density (pA/pF) in patient and control hiPSC-CMs (right). Values are mean ± standard error of the mean (SEM; *n* = 7 for patient and control). *P <0.05, versus control. **(B)** IKs measured in iCell Cardiomyocyte (Cellular Dynamics International Inc., Madison, WI, USA), family control and patient hiPSC-CMs treated with ML277 (left). Averaged tail current density (pA/pF) of iCell, family control and patient hiPSC-CMs (right). Values are mean ± SEM. *P <0.05, versus baseline. LQT1, type 1 long QT syndrome.

**Figure 5:**
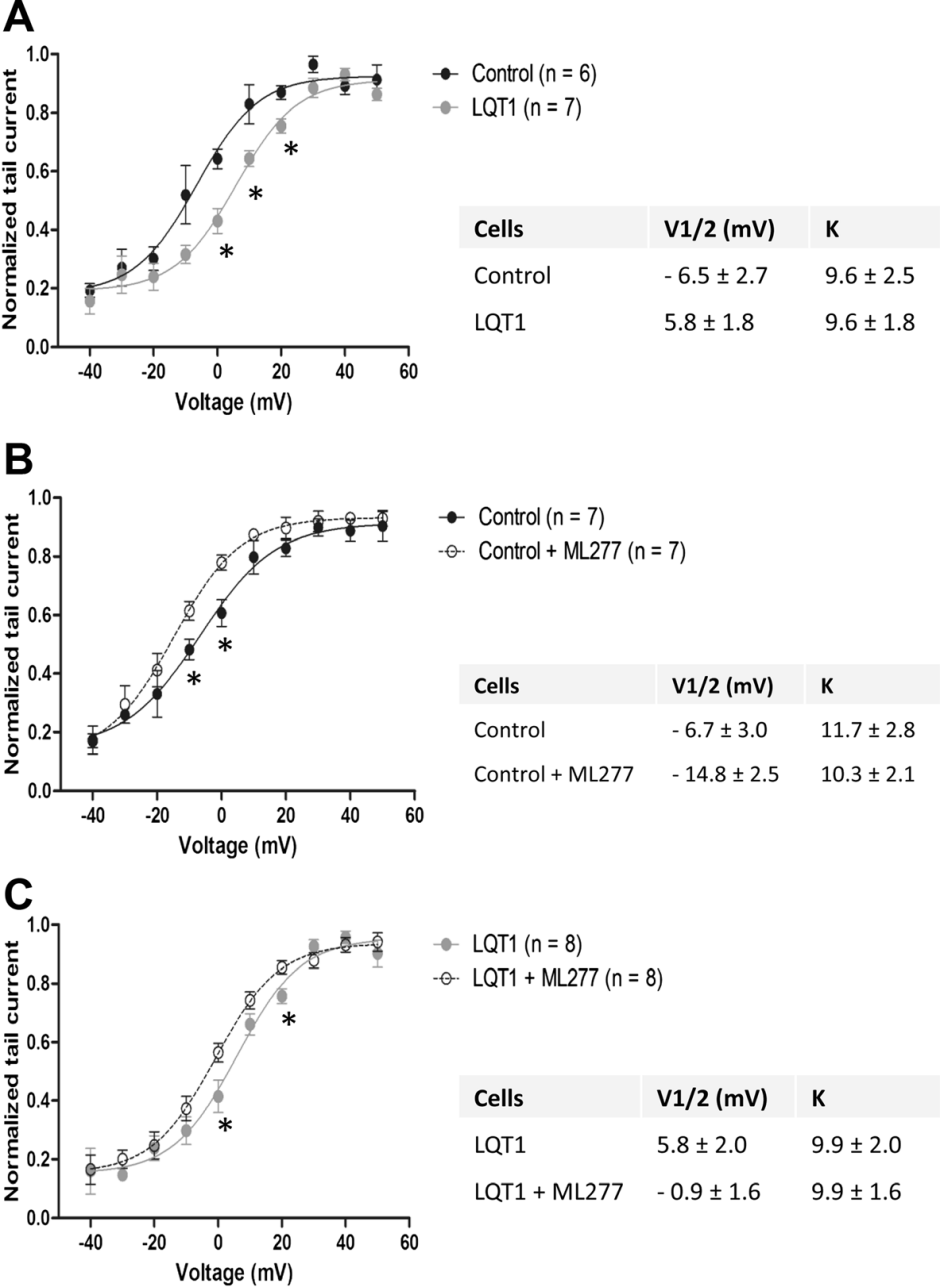
**Activation kinetics of slowly activating delayed rectifier potassium channel measured in human induced pluripotent stem cell cardiomyocytes. (A)** Activation kinetics of slowly activating delayed rectifier potassium channel (IKs) measured in control and patient human induced pluripotent stem cell cardiomyocytes (hiPSC-CMs). **(B)** Activation of IKs measured in family control hiPSC-CMs prior to and post ML277 treatment. **(C)** Activation of IKs measured in patient hiPSC-CMs prior to and post ML277 treatment. The half-maximal activation voltage (V(1/2)) was calculated and presented in the inserted tables. *P <0.05, versus control or baseline. LQT1, type 1 long QT syndrome.

**Figure 6:**
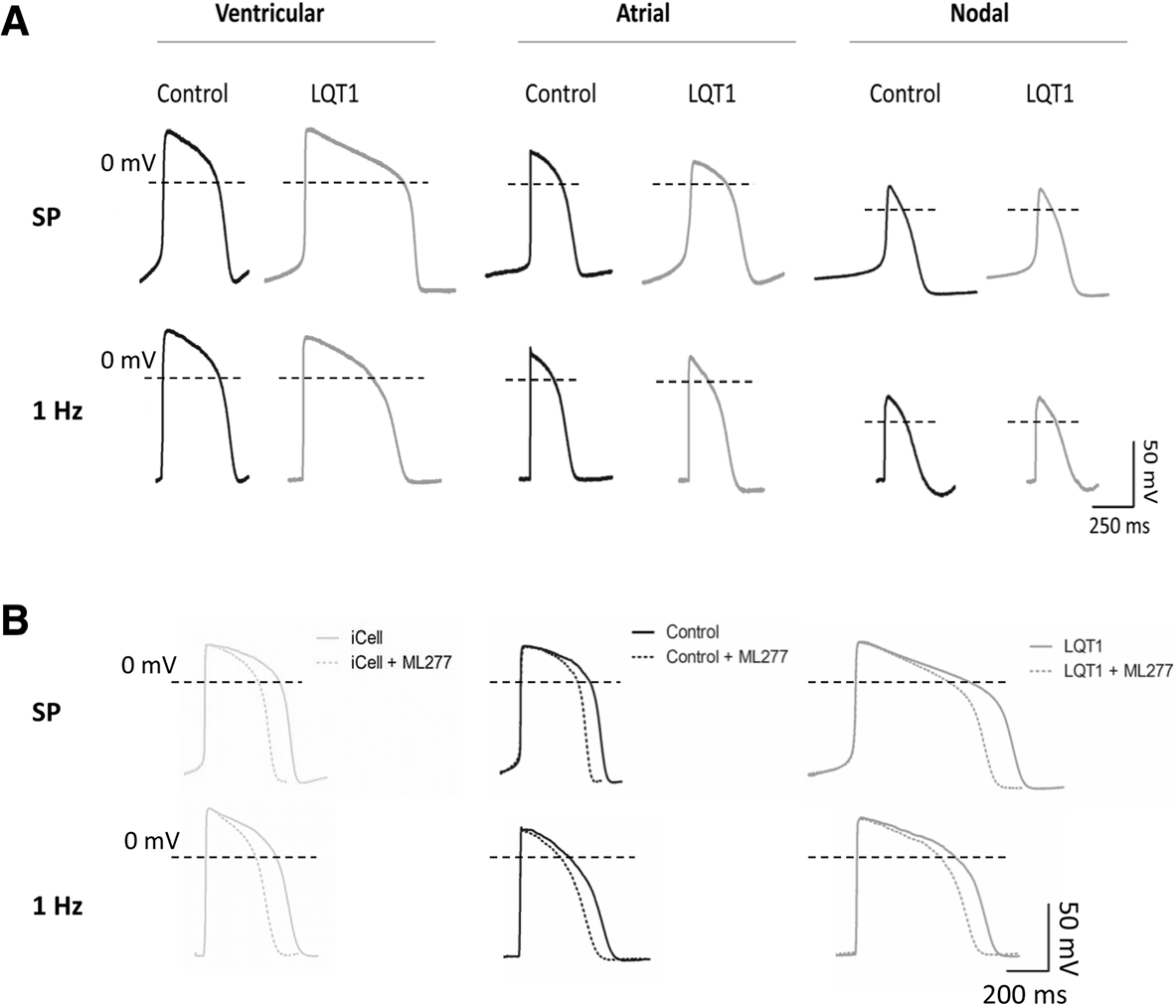
**Action potential properties of human induced pluripotent stem cell cardiomyocytes. (A)** Traces of spontaneous action potentials measured in ventricular-like, atrial-like, and nodal-like human induced pluripotent stem cell cardiomyocytes (hiPSC-CMs) derived from control and patient human induced pluripotent stem cells (hiPSCs; upper panel). Traces of paced (1 Hz) action potentials measured in ventricular-like, atrial-like, and nodal-like hiPSC-CMs derived from control and patient hiPSCs (lower panel*).* **(B)** Traces of spontaneous (upper panel) and paced (1 Hz) (lower panel) action potentials measured in iCell Cardiomyocyte (Cellular Dynamics International Inc., Madison, WI, USA), family control and patient ventricular-like hiPSC-CMs at baseline (solid lines) and post ML277 treatment (dashed lines). Horizontal dashed lines crossing the AP traces indicate a voltage of 0 mV. LQT1, type 1 long QT syndrome; SP, spontaneous.

Compared with the control, the hiPSC-CMs from the LQT1 patient demonstrated decreased IKs current (Figure 4A). The baseline level of IKs in patient hiPSC-CMs was significantly lower than that of the family control (Figure 4A). A positively shifted half-maximal activation voltage (V(1/2)) of IKs activation in patient hiPSC-CMs was noted compared with the control (Figure 5A), indicating a decreased activation of IKs. Moreover, prolonged action potential duration (APD) was observed in patient hiPSC-CMs of the ventricular-like subtype as the APD at 50% repolarization and the APD at 90% repolarization in patient hiPSC-CMs were ~1.85-fold that of the control (Figure 6A, Table 1).

**Table 1:** **Action potential parameters of ventricular-like human induced pluripotent stem cell cardiomyocytes**

**AP**	**APA (mV)**	**APD50 (ms)**	**APD90 (ms)**	**APD90/APD50**	**Overshoot (mV)**	**MDP (mV)**	**Heart rate (beats/minute)**
**Spontaneous**							
LQT1 patient (*n* = 41)	98.2 ± 1.3	603.9 ± 39.2***	671.0 ± 41.1***	1.12 ± 0.01	40.8 ± 1.0	−58.7 ± 1.1	69.4 ± 4.7
Control (*n* = 17)	99.1 ± 2.6	319.3 ± 13.8	372.9 ± 14.2	1.18 ± 0.02	40.0 ± 1.4	−59.8 ± 0.7	71.0 ± 5.2
**Paced (1 Hz)**							
LQT1 patient (*n* = 30)	100.4 ± 1.1	477.1 ± 23.9***	545.3 ± 26.6***	1.15 ± 0.02	42.2 ± 1.0	−58.3 ± 1.3	60
Control (*n* = 8)	101.5 ± 6.5	349.9 ± 23.7	397.5 ± 32.2	1.13 ± 0.02	42.4 ± 2.5	−58.3 ± 3.2	60

AP, action potential; APA, action potential amplitude; APD50, action potential duration at 50% repolarization; APD90, action potential duration at 90% repolarization; LQT1, type 1 long QT syndrome; MDP, maximum diastolic potential. ***P <0.005, versus control.

Our data show that patient-specific hiPSC-CMs have decreased amplitude and activation of IKs and shortened APDs, indicating that patient hiPSC-CMs faithfully recapitulated the typical cellular electrophysiological characteristics of LQT1.

Determined by the AP properties, it is noted that a majority (~70%) of the hiPSC-CMs from the LQT1 patient and control were ventricular-like while the rest were atrial-like or nodal-like hiPSC-CMs (Figure 6A, Table S2 in Additional file 1). All subtypes of hiPSC-CMs were capable of spontaneous contraction with a lower maximal diastolic potential. The ratio of the hiPSC-CM subtypes and the AP properties we observed are in close agreement with previous reports [1-4,17].

### *KCNQ1* overexpression in LQT1 patient hiPSC-CMs rescued the LQT1 phenotypes

Increased IKs current and shortened APD were observed in LQT1 patient hiPSC-CMs transfected with WT human *KCNQ1* (Figure S2 in Additional file 1). It was noted that the LQT1 phenotypes were not fully restored. This is probably because some of the green fluorescent protein-positive cells (cells were co-transfected with a green fluorescent protein vector) selected for patch-clamp assay may not contain WT *KCNQ1* vector. Nevertheless, our data support that the *KCNQ1* mutation in our patient is responsible for the LQT1 phenotypes.

### ML277 rescued the abnormal electrophysiological phenotypes of LQT1 in patient-specific hiPSC-CMs

**Figure 7:**
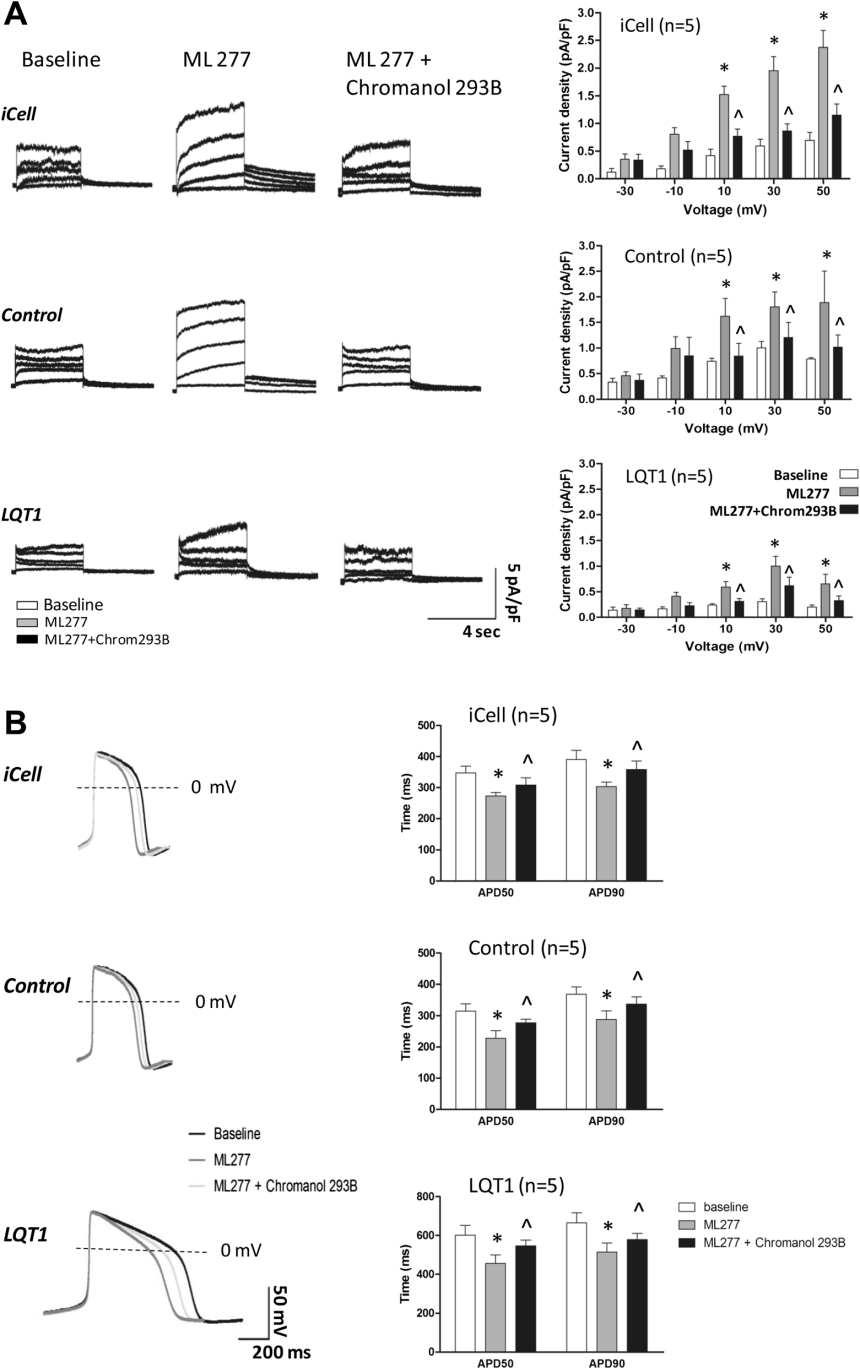
**Effects of Chromanol 293B on slowly activating delayed rectifier potassium channel and APD in ML277 treated human induced pluripotent stem cell cardiomyocytes. (A)** Effects of Chromanol 293B on slowly activating delayed rectifier potassium channel in ML277-treated iCell Cardiomyocyte (Cellular Dynamics International Inc., Madison, WI, USA), family control and LQT1 human induced pluripotent stem cell cardiomyocytes (hiPSC-CMs). **(B)** Effects of Chromanol 293B on action potential duration in ML277-treated iCell, family control and LQT1 hiPSC-CMs. *P <0.05, versus control; ^*P* <0.05, versus ML277 treated. APD50, action potential duration at 50% repolarization; APD90, action potential duration at 90% repolarization; LQT1, type 1 long QT syndrome; pA/pF, averaged peak and tail current density.

To preciously evaluate the effect of ML277, iCell – the normal control hiPSC-CM adopted by Yu and colleagues for assessing the effects of ML277 [16] – was included in this study as reference. Application of ML277 to iCell, the family control hiPSC-CMs and the LQT1 patient-specific hiPSC-CMs uniformly led to a significant elevation of IKs and the average IKs increments (at different voltages) were ~1.64-fold, 1.30-fold and 1.63-fold, respectively (Figure 4B). Moreover, a negatively shifted V(1/2) of IKs_,_ activation was observed in both family control and patient hiPSC-CMs treated with ML277 (Figure 5B, C), indicating that ML277 could enhance the voltage-gated activation of IKs_._ In corresponding to the elevation of IKs, it was noted that ML277 treatment shortened the APD (~20% reduction in APD at 50% repolarization and the APD at 90% repolarization) in all types of cells (Figure 6B, Table 2). Finally, the effect of ML277 was further validated by suppressing the effects of ML277 with Chromanol 293B (a specific IKs blocker) in iCell, family control and LQT1 patient hiPSC-CMs (Figure 7). Chromanol 293B abolished ML277-induced increased IKs and shortened the APD.

**Table 2:** **Action potential parameters of ventricular-like human induced pluripotent stem cell cardiomyocytes treated with ML277**

**Spontaneous APs**		**APA (mV)**	**APD50 (ms)**	**APD90 (ms)**	**APD90/APD50**	**Overshoot (mV)**	**MDP (mV)**	**Heart rate (beats/minute)**
LQT1 patient	Baseline (*n* = 11)	98.4 ± 1.7	601.1 ± 51.1	665.4 ± 51.9	1.12 ± 0.01	40.1 ± 2.4	−60.9 ± 1.6	64.6 ± 6.0
	ML277 (1 μM) (*n* = 11)	98.1 ± 1.3	455.5 ± 44.6**	514.7 ± 46.0**	1.15 ± 0.02	38.0 ± 2.8	−61.1 ± 1.7	66.9 ± 1.6
Control (father)	Baseline (*n* = 9)	99.1 ± 3.5	315.1 ± 23.1	368.8 ± 22.5	1.13 ± 0.01	41.5 ± 1.1	−59.4 ± 1.1	75.8 ± 6.8
	ML277 (1 μM) (*n* = 9)	99.2 ± 2.4	228.0 ± 24.4**	288.4 ± 27.4**	1.17 ± 0.02	40.7 ± 2.7	−60.6 ± 1.5	76.0 ± 8.0

AP, action potential; APA, action potential amplitude; APD50, action potential duration at 50% repolarization; APD90, action potential duration at 90% repolarization; LQT1, type 1 long QT syndrome; MDP, maximum diastolic potential. **P <0.01, versus baseline.

Our data indicate that ML277 is capable of reversing the electrophysiological phenotypes in LQT1 hiPSC-CMs by increasing the amplitude and enhancing the activation of IKs and shortening APDs. Yet the effects of ML277 were not limited to LQT1 patient hiPSC-CMs, which have compromised activating IKs currents, but also extended to normal hiPSC-CMs. However, it is noted that in both patient and control hiPSC-CMs treated with ML277, the changes in APDs were less closely correlated with the changes in IKs.

## Discussion

Our data provided sufficient evidence to support that the heterozygous deletion of exon 7 of *KCNQ1* in our LQT1 patient is pathogenic and contributes to the LQT1 phenotypes. Our patient hiPSC-CMs recapitulated the typical cellular electrophysiological phenotypes of LQT1 including reduced IKs current amplitudes and activation and prolonged APDs similar to those previously observed in the hiPSC-CMs derived from a LQT1 patient with a missense mutation (R190Q) [2].

Exon 7 of *KCNQ1* encodes a major part of the S6 transmembrane-spanning segments (the pole region) and the loop connecting S5 and S6. In LQT1 patient hiPSC-CMs, we found that the WT α-subunit of Kv7.1 is the predominant form, suggesting that the heterozygous deletion of exon 7 leads to haploinsufficiency associated with reduced IKs currents and shortened APDs. On the other hand, we found that ∆exon7 in the *KCNQ1* gene leads to three exon-skipping mRNAs all known to be capable of synthesis of truncated K_V_7.1 in COS7 lines, as demonstrated by Tsuji and colleagues who identified the same panel of exon-skipping *KCNQ1* mRNAs (Δexon 7, Δexon 8 and Δexon 7 + 8) in the blood cells of a LQT1 patient with heterozygous splice-site missense mutation in the last base of exon 7 (c.1032G > A) [18]. Similar to our study, Tsuji et al noted that normal individuals had minor fractions of splicing variants (Δexon 7 + 8, 0.1% of total *KCNQ1* transcripts; and Δexon 8, 6.9% of total *KCNQ1* transcripts) while the LQT1 patient had a remarkable increases of exon-skipping mRNAs (Δexon 7, 23.5%; Δexon 7 + 8, 16.8%; Δexon 8, 4.5%). Moreover, Tsuji and colleagues observed a membranous distribution pattern of the wild type Kv7.1 and a perinuclear localization pattern of the truncated proteins. However, they reported that all truncated Kv7.1 displayed no time-dependent IKs currents in Xenopus oocytes, indicating that those truncated proteins do not possess IKs channel function [18]. We believe that the truncated α-subunit of Kv7.1 proteins, which miss the pore region and imply a complete loss of function of the α-subunit included in the tetramer, may present in our patient-derived hiPSC-CMs at residual levels and serve as poison peptides leading to defective channel assemble with ancillary subunits and subsequently lead to the altered kinetics.

Trafficking deficiency of the alpha subunit of Kv7.1 can, in some cases such as the *KCNQ1*-T587M, a known trafficking mutation that caused haploinsufficiency, reduce localization of *KCNH2* (HERG) to the cell membrane [19]. This is due to a chaperone effect of *KCNQ1*-WT that helps to enhance the membrane localization of *KCNH2*. We measured the E4031 (a specific blocker of the rapid activating delayed rectifier potassium channel (IKr))-sensitive IKr density in hiPSC-CMs from the LQT1 patient and control, and observed no difference between them (data not shown). Moreover, in a latest report involving modeling of Jervell and Lange-Nielsen syndrome with patient hiPSC-CMs, the IKr tail density recorded in the hiPSC-CMs bearing a c.1781G > A *KCNQ1* mutation with a partial *KCNQ1* trafficking defect was found comparable with the WT control [20]. The impact of *KCNQ1* mutations on hERG might thus be mutation specific.

Current drug therapy for LQTS including LQT1 is hampered by a lack of specific and effective drugs that could specifically target the affected ion channels. Beta-blockers remain one of the few anti-arrhythmic drugs commonly used in LQTS treatment, yet their effects are limited and controversial. By high-throughput screening of the NIH Molecular Libraries Small Molecule Repository collection of over 300,000 compounds using an automated patch-camp system, a small molecule ML277 has been identified as a novel, potent and selective K_V_7.1 potassium channel activator [16,21]. Closely resembling the findings from Yu and colleagues [16], we demonstrated that ML277 increased IKs by 1.3-fold to 1.64-fold and shortened APD by ~20% in iCell as well as in LQT1 patient and family control hiPSC-CMs. The enhanced IKs in all cell groups is most likely to be achieved via the WT (functional) K_V_7.1 channels. The effect of ML277 in enhancing the amplitude of IKs as well as accelerating the activation rate (Figure 5) was further validated in a recent report [22] in which ML277 enhanced the IKs amplitude and shortened the APD in the adult guinea pig and canine ventricular myocytes. In the latter, ML277 also altered the gating kinetics by negatively shifting the V(1/2). The effect of ML277 on IKs activation is species specific [22]. It is believed that ML277 inhibits the voltage-dependent inactivation kinetics of *KCNQ1* to contribute to the augmentation of current amplitude while the hyperpolarizing shift of V(1/2) could significantly increase the maximal conductance [16].

ML277 could become a potential new and targeted drug for LQT1 without causing arrhythmia in normal individuals. The action of ML277 depends on the unsaturated status of the α-subunits of Kv7.1 channel with E1 subunits [16]. The association of KCNE1 with *KCNQ1* may thus prevent ML277 from triggering an arrhythmogenic effect in healthy individuals. Given the rapid response of hiPSC-CMs to ML277, we believe that the effects of ML277 are most probably achieved via directly effects on the Kv7.1 channel rather than affecting the synthesis and/or the trafficking of K_V_7.1. This notion is confirmed in a recent report which indicated that ML277 activates IKs by binding to an intersubunit space and allosterically influencing pore conductance and gating transitions [23].

The inward rectifier potassium channel (IK1) current plays an important role in determining the APD in cardiomyocytes in addition to the L-type calcium current (ICa,L), IKs and IKr. Absence of IK1 in hiPSC-CMs leads to inactivation of the cardiac transient outward potassium current (Ito) and loss of the phase 1 notch in an AP [23], which could lead to more activated L-type calcium channel and increased ICa,L [24] and prolonged phase 2 repolarization. We noted that cardiomyocytes derived from hiPSCs have a more depolarized status than adult cardiomyocytes characterized by a lower resting membrane potential (~60 to −50 mV) and higher automaticity (Figure 6). Such differences are associated with lack of IK1 current, as highlighted by Bett and colleagues and by Clusin [25,26]. Although the impacts on IKs and IKr currents and APD in hiPSC-CMs might be relatively limited, lack of IK1 current could alter the cardiac ion currents and APs and compromise the value of hiPSC-CMs in disease modeling and drug testing. As cardiac APs are shaped by several different outward currents (IK1_,_ ICa,L, IKs and IKr) with potential interactions or interplay among them, it is not surprising to notice that changes in IKs in hiPSC-CMs may not be proportional to that of APD.

In this study, we did not consider the impacts of lack of IK1 current on the APs of hiPSC-CMs and we did not evaluate the real-time contribution of cardiac ion currents such as IKs and IKr on the APs in beating cardiomyocytes. For precise disease modeling and more accurate drug testing, it might be valuable, in future studies, to restore IK1 current in hiPSC-CMs to produce more physiological relevant APs and to perform real-time measurement of cardiac ion currents (such as ICa,L_,_ IKs and IKr) concurrently with cardiac APs. Technologies such as the dynamic AP clamp [25,26] could be adopted to restore IK1 currents in hiPSC-CMs. Moreover, the efficacy and safety of ML277 could be tested in animal models.

Our data demonstrate that multiple factors can contribute to the pathology or act to correct LQTS. LQT1 patient hiPSC-CMs show decreased IKs amplitude and altered gaiting kinetics with the V(1/2) shifted in the hypopolarizing direction. ML277, on the other hand, enhanced the amplitude of IKs as well as restored the kinetic changes by accelerating the activation rate. Following the example of ML277, we believe that more candidate drugs targeting multiple factors could be identified for the potential therapy of LQT1.

## Conclusions

From a LQT1 patient, we identified a novel heterozygous exon 7 deletion mutation in the *KCNQ1* gene that leads to potential haploinsufficiency and trafficking defect. We generated hiPSC-CMs which faithfully recapitulated the typical LQT1 phenotypes. We found that the K_V_7.1 activator ML277 could partially rescue the electrophysiological phenotypes of LQT1 and may have therapeutic potential.

## Additional file

Additional file 1:
**A supplemental document describing supplemental methods and results.**

## Abbreviations

AP: action potential
APD: action potential duration
ECG: electrocardiogram
hiPSC-CM: human induced pluripotent stem cell cardiomyocyte
hiPSC: human induced pluripotent stem cell
ICa,L: L-type calcium current
IK1: inward rectifier potassium channel
IKr: rapid activating delayed rectifier potassium channel
IKs: slowly activating delayed rectifier potassium channel
*KCNQ1*: potassium channel, voltage-gated KQT-like subfamily Q, member 1
LQTS: long QT syndrome
LQT1: type 1 long QT syndrome
V(1/2): half-maximal activation voltage
WT: wild type
