# Supplementary material for: Characterization of a novel KCNQ1 mutation for type 1 long QT syndrome and assessment of the therapeutic potential of a novel IKs activator using patient-specific induced pluripotent stem cell-derived cardiomyocytes
Source: Stem Cell Res Ther. 2015 Mar 19;6(1):39. doi: 10.1186/s13287-015-0027-z (PMC4396080; doi:10.1186/s13287-015-0027-z)
Supplement: Additional file 1: — A supplemental document describing supplemental methods and results. [file 13287_2015_27_MOESM1_ESM.doc]

**Characterization of a novel KCNQ1 mutation for type 1 Long QT syndrome and assessment of the therapeutic potential of a novel *I*_ks_ activator using patient-specific induced pluripotent stem cell-derived cardiomyocytes**

Dongrui Ma, Heming Wei, Jun Lu, Dou Huang, Zhenfeng Liu, Li Jun Loh, Omedul Islam, Reginald Liew, Winston Shim and Stuart A Cook

**SUPPLEMENTAL MATERIAL**

**Supplemental Methods:**

**Mutation analysis**

Genomic DNA was isolated from the peripheral blood of LQT1 patient and control. All exons with flanking intronic sequences of the KCNQ1 gene were sequenced after PCR amplification and compared to the reference sequence. Other cardiac ion channel genes (49 in total) including KCNH2, SCN5A, CACNA1C (the genes responsible for LQT2, LQT3 and LQT8 or Timothy Syndrome) and KCNE1 (encodes the β-subunits of Kv7.1 channel) were screened.

**Generation and characterization of hiPSCs**

*Derivation of dermal fibroblasts*- Skin dermis from biopsy samples was manually separated, minced and plated onto a gelatin-coated petri-dish with Dulbecco’s modified eagle medium (DMEM, Invitrogen, USA) supplemented with 10% fetal bovine serum. Fibroblasts migrated out of the dermis tissue within 7 days and were sub-cultured till passage 2 for reprogramming.

*Generation and characterization of hiPSCs-* Dermal fibroblasts were reprogrammed into hiPSCs via transduction of retroviral vectors containing OCT-4, SOX-2, KIF-4, and c-Myc . In brief, control and patient hiPSC lines (4 line each) with high stability and consistent cardiac differentiation efficiency were cultured in mTeSR™1 Medium (STEMCELL Technologies, Inc) on Matrigel® (BD Bioscience)-coated culture petri-dishes. Cells were passaged with StemPro® Accutase® Cell Dissociation Reagent (Life Technologies). hiPSCs were characterization as previously described . The pluripotency of hiPSCs was confirmed by their expression of human pluripotent stem cell markers determined by immunofluorescence staining and further validated by teratoma formation assay.

*Immunofluorescence staining of human pluripotent markers-* hiPSCs were fixed with 4% paraformaldehyde in PBS (phosphate buffered saline) for 15 min at 4°C. After washing with the cells and EBs were treated with 0.2% Tween-20 in PBS for 5 min at room temperature. The cells were then blocked with PBS containing 5% goat serum for 30 min at room temperature. Primary monoclonal antibodies included Oct-4 (Milipore, USA) diluted 1:500; SSEA-4 (Milipore, USA) diluted 1:500; Tra-1-60 (Milipore, USA) diluted 1:500; and Tra-1-81 (Milipore, USA) diluted 1:500. Secondary antibodies used were Alexa568-conjugated goat anti-mouse IgM (1:500), Alexa568-conjugated goat anti-mouse IgG (1:500), cell nuclei were counter stained with DAPI (Invitrogen, USA). Fluorescent images were viewed by a Laser Scanning Confocal Microscopy (Carl Zeiss LSM 710).

*Teratoma formation assay*- A total of 2 X 10^6^ of hiPSCs was injected into the kidney capsule of NOD-SCID mice. After 12 weeks, tumors were explanted, fixed and paraffin embedded. Dissected sections were stained with haematoxylin and eosin .

**Cardiac differentiation and preparation of hiPSCs**

Cardiac differentiation of hiPSCs was achieved via Wnt signaling inhibition as described previously . The hiPSC line with best and consistent cardiac efficiency was adopted in this study. Five weeks after cardiac differentiation, the contracting cardiomyocytes portions were dissected and collected for Rt-PCR and Western blot assays . For patch-clamp assay and immunofluorescence staining, the contracting cardiomyocyte portions were dissociated into single cardiomyocytes and plated on 3.5 cm gelatin-coated regular or glass bottom petri-dishes .

**The expression of KCNQ1 and K_V_7.1**

Quantitative RT-PCR (qRT-PCR) assay for the expression of KCNQ1 was performed with RNA isolated from contracting hiPSC-CMs clusters (manually dissected and trimmed from the non-contraction portions) from patient and control. Total RNA was transcribed into cDNA. PCR primers were shown below in **Table S1**. The KCNQ1 PCR was run for 42 cycles and the GAPDH PCR was run for 29 cycles. Sanger sequencing was performed on gel purified PCR products of KCNQ1 of various sizes.

**Table S1 PCR primers**

| **Gene access #** | **Gene** | **SEQUENCE (5’ to 3’) of PCR PRIMERS** | |
| --- | --- | --- | --- |
| NM_000218.2 | KCNQ1 (exon 6 - exon 9) | F | GTA CCT GGC TGA GAA GGA CG |
|  |  | R | AGA CTT CTT GGG TTT GGG GC |
| NM_002046.5 | GAPDH | F | CCT GAA CCC TAA GGC CAA CCG |
|  |  | R | GCT CAT AGC TCT TCT CCA GGG |

**Determination of the total levels and intracellular localization of Kv7.1**

The total cellular K_V_7.1 level was determined by Western Blot. Cell lysate was prepared from the clusters of hiPSC-CMs. Proteins were separated by 10% SDS/PAGE under denaturing conditions and transferred to a PVDF membrane. The membrane was subsequently incubated with primary antibody (Anti-K_V_7.1/KCNQ1, 1:500, Alomone Labs, Jerusalem, Israel; Anti-GAPDH, 1:1000, Cell Signaling, MA, USA) overnight in 4°C. After washing with TBST, the membrane was incubated with rabbit peroxidase-conjugated secondary antibody (1:5000) at room temperature for 1 h, and developed with WesternBright HRP substrate (Advansta, USA).

The intracellular localization of K_V_7.1 in hiPSC-CMs was determined by immunofluorescence assay. hiPSC-CMs were fixed with 4% paraformaldehyde at room temperature for 15 min and permeabilized with 0.1% Triton X-100 for 10 min at room temperature. The cells were then incubated with primary antibody including anti-Kv7.1 (Anti-K_V_7.1/KCNQ1, 1:200, Alomone Labs, Jerusalem, Israel); anti-α-actinin (clone EA-35, Sigma, St Louis, USA) anti-Golgi (Anti-Golgin 97, 1:200, Life Technologies, Singapore) overnight at 4 °C, followed by secondary antibody at room temperature for 1 h. The cells were then mounted with DAPI and examined under confocal microscope. The immunofluorescence intensity was quantified with ImageJ software, where signals with distance to nucleus less than half of radius were marked as perinuclear and the rest marked as membranous.

**Over expression of human KCNQ1 in LQT1 patient hiPSC-CMs**

TransIT®-LT1 Transfection Reagent (Mirus Bio LLC, Madison, USA) reported to have an transcription efficiency ( ~ 70%) in iCell®Cardiomyocytes was adopted. WT human KCNQ1expression vector was transiently co-transfected with a GFP vector into LQT1 patient hiPSC-CMs dissociated and plated in 3.5 cm gelatin-coated petri-dishes. Around ~50 % of GFP(+) cells were observed between 48~72 hours post transfection. GFP (+) cells were subjected to whole cell patch clamp assay to measure APs and *I*_Ks_ currents.

**Whole cell patch-clamp recordings**

All cardiomyocytes used in this study were 6~7 weeks post cardiac differentiation to ensure that they maintained a similar maturity. Axon patch 200B (Axon Instruments, Sunnyvale, USA) was used for whole cell patch clamp recordings and data acquisition were controlled by Axon Instruments pClamp10 software via a Digidata 1440 acquisition system. The cell size of hiPSC-CMs was determined by cell membrane capacitance (Cm(pF)(Cm=πd2/100) ) automatically measured by the Patch-Clamp sampling software（Clampex） during whole cell current recording.

*Current-clamp protocol for action potentials measurements-* Under current-clamp mode, action potentials (APs) of hiPSC-CMs were recorded with whole cell patch-clamp configuration in Tyrode’s solution (in mM): 140 NaCl, 5.4 KCl, 1.8 CaCl_2_, 1 MgCl_2_, 10 Hepes, 10 glucose, pH 7.4 (NaOH) at 37^o^C. The glass pipette was filled in with the pipette solutions (in mM): 50 KCl, 80 K-Asparatate, 1 MgCl_2_, 3 MgATP, 10 HEPES, pH 7.4 (KOH). Experiments were performed at 34~37°C.

Spontaneous APs were recorded in spontaneously contracting hiPSC-CMs. Paced APs were recorded in hiPSC-CMs elicited at 1 Hz by 3 ms depolarizing current pulse at approximately twice the threshold (1.2× threshold) at a cycle length of 1s (1Hz) through the patch pipette. Action potentials were low pass filtered at 2 kHz and sampled at 20 kHz. The average action potential duration (APD) at 90%, 70%, 50% and 20% of repolarisation (APD90, APD70, APD50 and APD20), AP amplitude (APA), maximal upstroke velocity (dV/dt_max_), contraction rate and resting membrane potential (BMP) were determined. The ventricular-like (V), atrial-like (A), and nodal-like (N) hiPSC-CMs were identified by their characteristic AP properties including APA, APD, and dV/dt_max_ .

*Voltage-clamp protocols- I*_ks_ currents were measured under the voltage-clamp mode at room temperature. The average series resistance was 1 to 1.5 MΩ before compensation (70% to 80%) and the voltage error was ~2.0 mV. Currents were low-pass filtered at 5 kHz and digitized at a sampling rate of 5 kHz (Digidata 1440, Axon Instruments). For *I*_ks_ current density (pA/pF), currents were normalized to the cell capacitance and expressed in pA/pF. The voltage dependence of KCNQ1 channel activation was measured. Activation curves were determined by fitting a Boltzmann expression to calculated conductance (Gv) values. The equation used was: *Gv* 5 *G*max/{1 1 exp [2(*V* 2 *V*1/2)/*k*]}. *G*max is the maximum conductance, *V is* the membrane voltage, *V*1/2 is the half activation voltage, and *k is* the slope factor. Experiments were performed at 37°C.

**Supplemental data:**

**Table S2. The AP parameters of hiPSC-CMs**

| **Spontaneous APs** | **CM subtypes** | **Cells (n)** | **APA (mV)** | **APD50 (ms)** | **APD90 (ms)** | **APD90/APD50** | **Overshoot (mV)** | **MDP (mV)** | **Heart Rate (beats/min)** |
| --- | --- | --- | --- | --- | --- | --- | --- | --- | --- |
| LQT1  patient | Ventricular | 41 | 98.2 ± 1.3 | 603.9 ± 39.2*** | 671.0 ± 41.1*** | 1.12 ± 0.01 | 40.8 ± 1.0 | -58.7 ± 1.1 | 69.4 ± 4.7 |
|  | Atrial | 9 | 86.7 ± 2.4 | 217.3 ± 21.6 | 307.5 ± 33.5 | 1.31 ± 0.05 | 33.1 ± 2.1 | -55.9 ± 2.6 | 95.8 ± 9.4 |
|  | Nodal | 4 | 72.7 ± 3.7 | 148.1 ± 16.0 | 232.9 ± 5.5 | 1.57 ± 0.08 | 31.1 ± 0.8 | -41.9 ± 5.0 | 74.0 ± 8.2 |
| Control  (Father) | Ventricular | 17 | 99.1 ± 2.6 | 319.3 ± 13.8*** | 372.9 ± 14.2*** | 1.18 ± 0.02 | 40.0 ± 1.4 | -59.8 ± 0.7 | 71.0 ± 5.2 |
|  | Atrial | 4 | 88.8 ± 7.9 | 167.1 ± 26.1 | 222.6 ± 30.8 | 1.33 ± 0.06 | 34.6 ± 8.1 | -55.9 ± 2.6 | 94.5 ± 2.6 |
|  | Nodal | 4 | 72.1 ± 2.2 | 153.0 ± 17.4 | 245.3 ± 12.9 | 1.47 ± 0.05 | 30.1 ± 1.2 | -42.7 ± 1.2 | 69.5 ± 7.4 |
|  | | | | | | | | | |
| **Paced APs ( 1 Hz)** | **CM subtypes** | **Cells (n)** | **APA (mV)** | **APD50 (ms)** | **APD90 (ms)** | **APD90/APD50** | **Overshoot (mV)** | **MDP (mV)** | **Heart Rate (beats/min)** |
| LQT1 patient | Ventricular | 30 | 100.4 ± 1.1 | 477.1 ± 23.9** | 545.3 ± 26.6** | 1.15 ± 0.02 | 42.2 ± 1.0 | -58.3 ± 1.3 | 60 |
|  | Atrial | 6 | 86.8 ± 2.6 | 197.1 ± 3.8 | 246.4 ± 10.1 | 1.25 ± 0.04 | 31.2 ± 4.1 | -55.8 ± 2.9 | 60 |
|  | Nodal | 6 | 71.0 ± 4.2 | 158.8 ± 3.8 | 248.6 ± 18.5 | 1.56 ± 0.05 | 30.9 ± 3.2 | -41.2 ± 3.7 | 60 |
| Control  (Father) | Ventricular | 8 | 101.5 ± 6.5 | 349.9 ± 23.7 | 397.5 ± 32.2 | 1.13 ± 0.02 | 42.4 ± 2.5 | -58.3 ± 3.2 | 60 |
|  | Atrial | 0 | _ | _ | _ | _ | _ | _ | _ |
|  | Nodal | 3 | 70.5 ± 2.1 | 143.2 ± 1.3 | 237.8 ± 17.8 | 1.66 ± 0.09 | 30.8 ± 2.3 | -42.2 ± 2.6 | 60 |

APA: action potential amplitude. APD50: action potential duration at 50% repolarization. APD90: action potential duration at 90% repolarization. MDP: maximum diastolic potential. ** p < 0.01, vs. control. *** p < 0.005, vs. control.


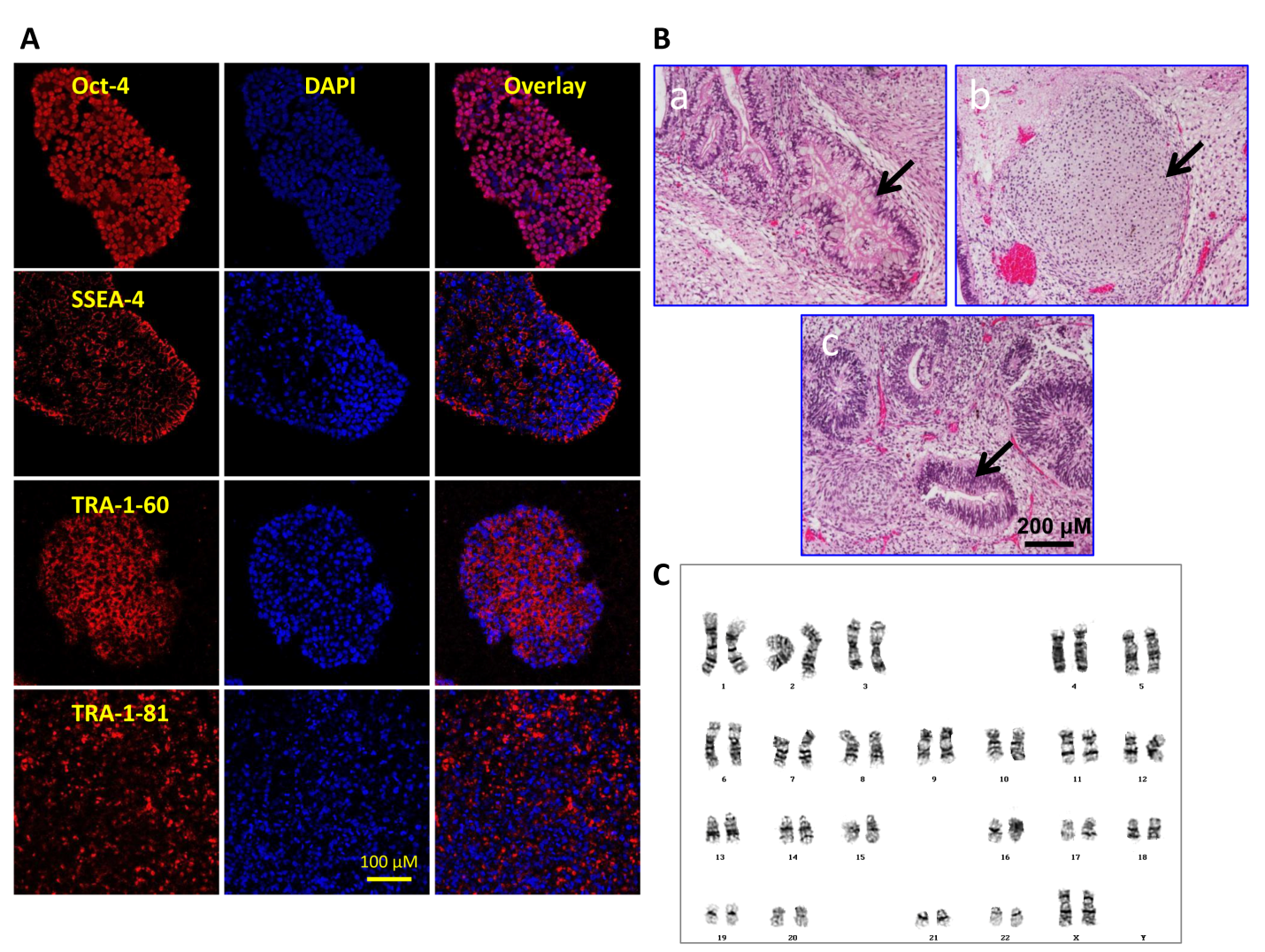


**Figure S1. Characterization of hiPSCs. (A**) Representative immunofluorescence images of patient hiPSC colonies stained positive for pluripotent stem cell markers Oct-4, SSEA-4, Tra-1-60 and Tra-1-81. Scale bar: 100μM. (**B**) Representative images of HE-stained sections of teratoma formed in SCID mice injected with LQT1 patient-derived hiPSCs. Characteristic tissues representing three germ layers are shown as intestine for endoderm (*a***)**; cartilage for mesoderm (*b***)**; and neuroepithelium for ectoderm (*c*). Scale bar: 200μM. (C) The karyotype of the hiPSCs derived from the LQT1 patient.


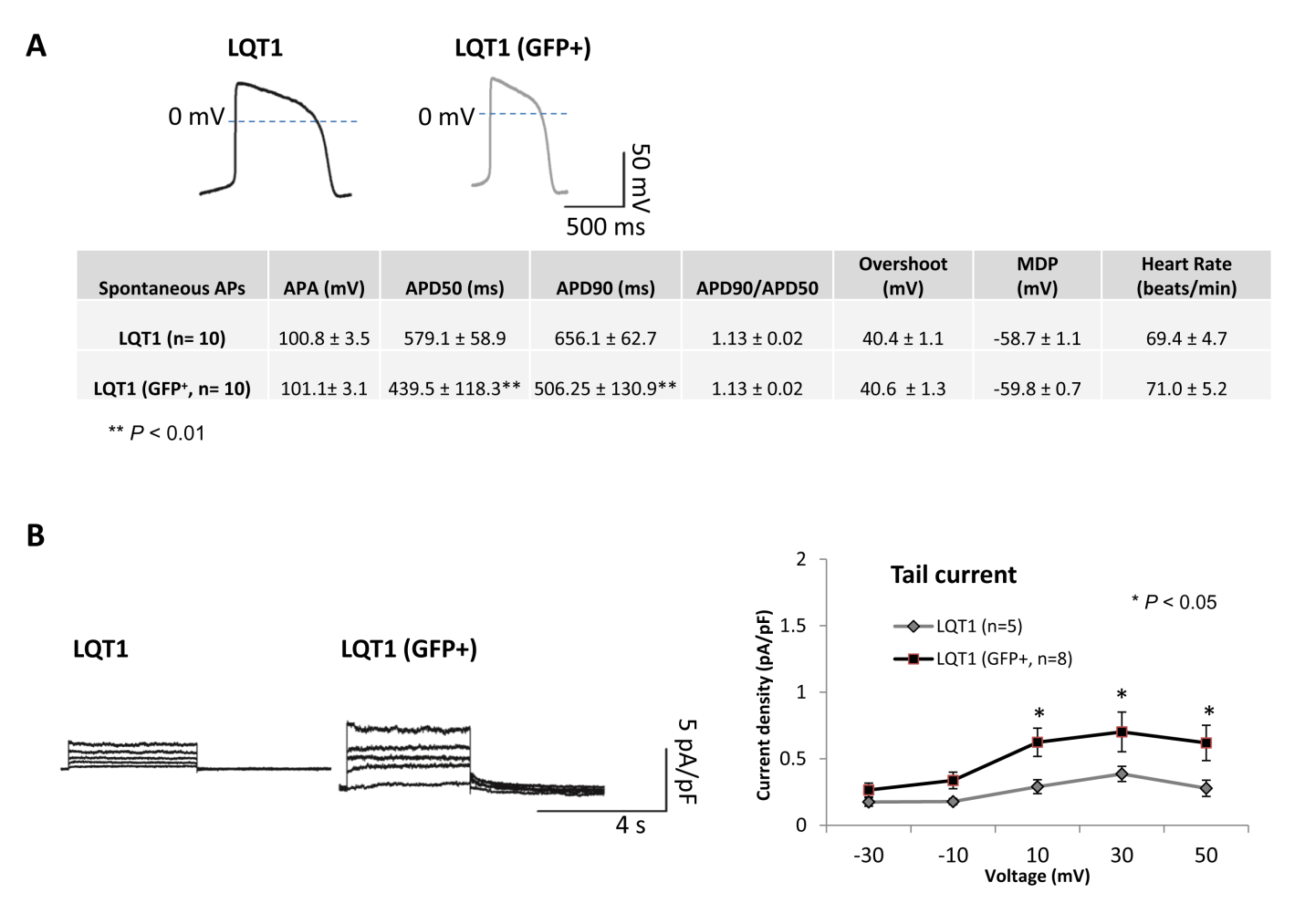


**Figure S2. KCNQ1 over expression rescued the LQT1 phenotypes in LQT1 patient hiPSC-CMs (A**) Representative AP traces recorded in LQT1 patient hiPSC-CMs without and with co-transfection of KCNQ1 and GFP vectors (Top panel). The bottom table shows the AP properties. (**B**) Representative *I*_Ks_ currents traces recorded in LQT1 patient hiPSC-CMs without and with co-transfection of KCNQ1 and GFP vectors (Left). The *I*_Ks_ current tail density was plotted and presented (Right).

REFERENCES

[1] Takahashi K, Tanabe K, Ohnuki M, Narita M, Ichisaka T, Tomoda K, et al. Induction of pluripotent stem cells from adult human fibroblasts by defined factors. Cell. 2007;131:861-72.

[2] Ma D, Wei H, Lu J, Ho S, Zhang G, Sun X, et al. Generation of patient-specific induced pluripotent

stem cell-derived cardiomyocytes as a cellular model of arrhythmogenic right ventricular

cardiomyopathy. European heart journal. 2013;34:1122-33.

[3] Lian X, Hsiao C, Wilson G, Zhu K, Hazeltine LB, Azarin SM, et al. Robust cardiomyocyte differentiation from human pluripotent stem cells via temporal modulation of canonical Wnt signaling. Proc Natl Acad Sci U S A. 2012;109:E1848-57.

[4] Ma J, Guo L, Fiene SJ, Anson BD, Thomson JA, Kamp TJ, et al. High purity human-induced pluripotent stem cell-derived cardiomyocytes: electrophysiological properties of action potentials and ionic currents. American journal of physiology Heart and circulatory physiology. 2011;301:H2006-17.

[5] Hoekstra M, Mummery CL, Wilde AA, Bezzina CR, Verkerk AO. Induced pluripotent stem cell derived cardiomyocytes as models for cardiac arrhythmias. Frontiers in physiology. 2012;3:346.
